# SNOMED CT entity linking challenge

**DOI:** 10.1093/jamia/ocaf104

**Published:** 2025-07-14

**Authors:** Rory Davidson, Will Hardman, Guy Amit, Yonatan Bilu, Vincenzo Della Mea, Aleksandr Galaida, Irena Girshovitz, Mikhail Kulyabin, Mihai Horia Popescu, Kevin Roitero, Gleb Sokolov, Chen Yanover

**Affiliations:** SNOMED International, London W2 6BD, United Kingdom; Veratai Ltf, Woking GU22 7QW, United Kingdom; KI Research Institute, Kfar Malal, Israel; KI Research Institute, Kfar Malal, Israel; Department of Mathematics, Computer Science and Physics, University of Udine, Udine 33100, Italy; Grid Dynamics, Yerevan 0023, Armenia; KI Research Institute, Kfar Malal, Israel; VisioMed.AI, Moscow 125212, Russia; Department of Mathematics, Computer Science and Physics, University of Udine, Udine 33100, Italy; Department of Mathematics, Computer Science and Physics, University of Udine, Udine 33100, Italy; VisioMed.AI, Moscow 125212, Russia; KI Research Institute, Kfar Malal, Israel

**Keywords:** SNOMED CT, entity linking, terminology, artificial intelligence

## Abstract

**Objective:**

This paper presents the results from a competition challenging participants to develop entity linking models using a subset of annotated MIMIC-IV-Note data and the SNOMED CT Terminology.

**Materials and Methods:**

As a basis for this work, a large set of 74 808 annotations was curated across 272 discharge notes spanning 6624 unique clinical concepts. Submissions were evaluated using the mean Intersection-over-Union metric, evaluated at the character level with the 3 best performing solutions awarded a cash prize.

**Results:**

The winning solutions employed contrasting approaches: a dictionary-based method, an encoder-based method, and a decoder-based method.

**Discussion:**

Our analysis reveals that concept frequency in training data significantly impacts model performance, with rare concepts proving particularly challenging. High concept entropy and annotation ambiguity were also associated with decreased performance.

**Conclusion:**

Findings from this work suggest that future projects should focus on improving entity linking for rare concepts and developing methods to better leverage contextual information when training examples are scarce.

## Background and significance

Much of the world’s healthcare data are stored in free-text documents, for example in patient notes written by clinicians. These data can be challenging to analyze. Clinical terminologies like SNOMED CT enable healthcare actors to map free-text to medical concepts, enabling the records to be unambiguously analyzed by computers.^1^ The process of identifying clinical concepts in free-text is called clinical coding or (as here) entity linking because it involves identifying the entities and linking them to a particular concept in a medical terminology.

The SNOMED Entity Linking challenge took place between December 2023 and March 2024. It was sponsored by SNOMED International and hosted on the DrivenData platform (https://www.drivendata.org/competitions/258/competition-snomed-ct/). Teams were tasked with training entity linking models to annotate discharge notes taken from MIMIC-IV-Note^2^ using the SNOMED Terminology. “Ground truth” annotations had been produced by a team of clinical annotators, resulting in 74 808 instances of 6624 clinical concepts across 272 documents.

The competition attracted 553 registered entrants of whom the top 3 won a prize. The 3 winning teams, KIRI, SNOBERT and MITEL-UNIUD, submitted different approaches: a dictionary-based method, an encoder-based method, and a decoder-based method, respectively.

## Objective

In this work, we perform a comparative analysis of the winning approaches and identify the most challenging factors affecting clinical entity linking models. We share the lessons learned and provide actionable recommendations to guide other researchers. Further details on the competition are available at https://www.drivendata.org/competitions/258/competition-snomed-ct/page/817/.

## Materials and methods

### Dataset

Prior works developing clinical entity linking datasets have focused on a restricted number of target concepts,^3^^,^^4^ on texts not generated within a clinical setting,^5^^,^^6^ or on texts that are not publicly available.^7^ We determined, therefore, to develop a new dataset which:

- Could be released into the public domain
- Used a wide range of target concepts
- Was large-scale
- Used real-world clinical notes

The data were sourced from MIMIC-IV-Note,^2^ hosted on the PhysioNet platform^8^ by the MIT Laboratory for Computational Physiology and available to accredited researchers who have completed Human Specimens training.

MIMIC-IV-Note contains 331K deidentified discharge notes for patients admitted to the Beth Israel Deaconess Medical Centre in Boston, MA. Records are structured into 7 sections (eg, “Chief Complaint,” “Physical Exams”). Most content is free-text containing clinical reasoning, assessments and treatment plans. Additionally, there are structured elements (eg, “Laboratory results,” “Physical exams”). The range of symptoms and treatments discussed is highly diverse. Furthermore, the records are rife with medical terminology, abbreviations and different writing styles. In sum, MIMIC-IV-Note constitutes a challenging dataset for machine learning applications.

SNOMED International recruited 6, geographically diverse annotators with clinical backgrounds to develop the “ground truth” dataset. Working on a randomly selected subset of MIMIC-IV-Note, the team spent 4 weeks annotating the dataset using the MedCatTrainer software.^9^

**Figure 1.**
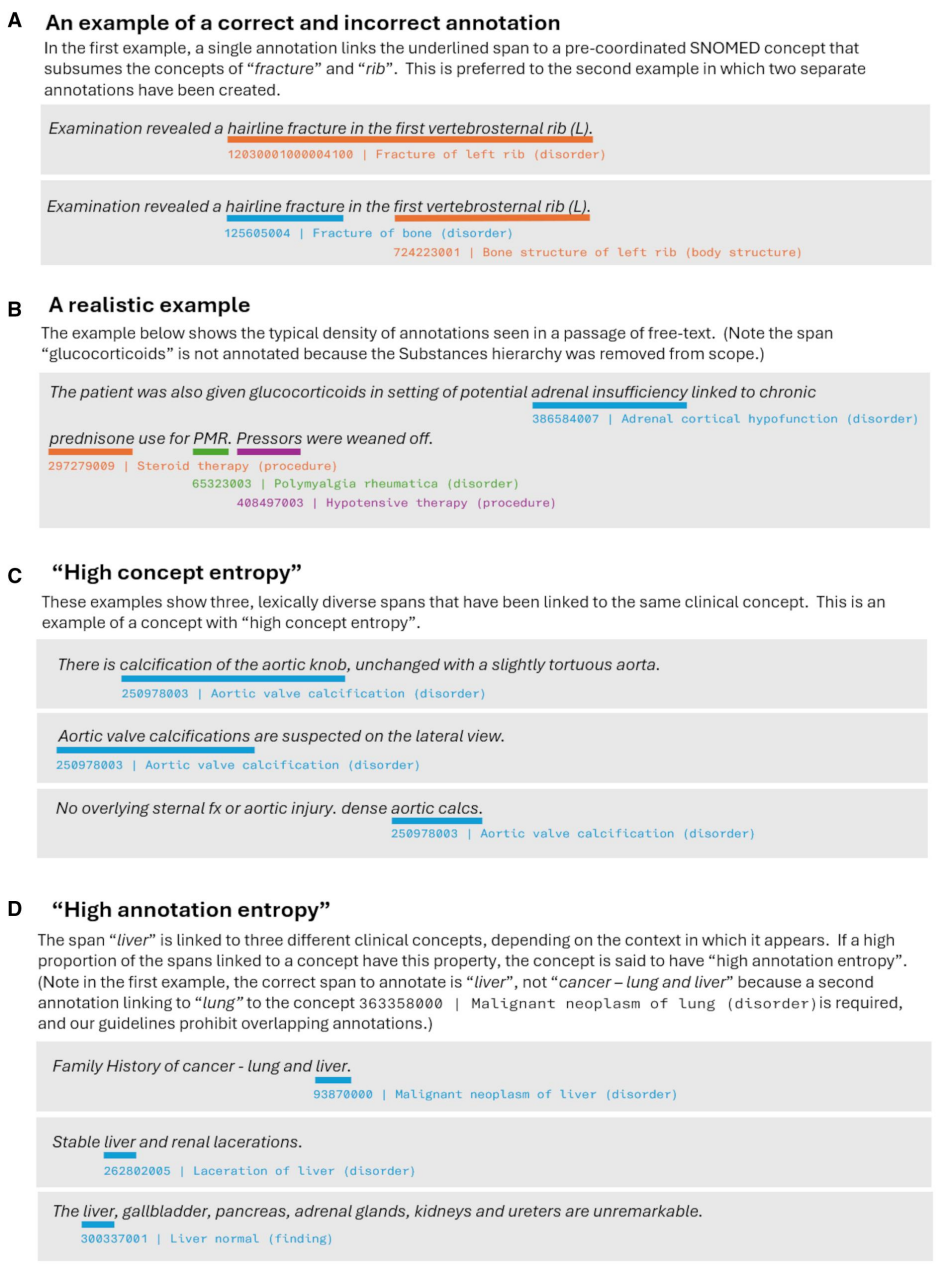
Example clinical sentences and annotations.

The annotators worked according to a policy document^10^ designed to facilitate consistent practice. Some of the key policies are summarized below:

1. **No post-coordination**. Due to the impracticalities of modeling all possible clinical concepts, SNOMED CT allows for primitive concepts to be combined to express more specific, clinical expressions. We did not require our annotators to post-coordinate, only to match spans to the closest SNOMED concept.
2. **Prefer pre-coordinated concepts to primitive concepts**. Commonly used clinical concepts—which are themselves compounds of more primitive concepts—are often directly modeled in SNOMED CT to facilitate easy usage. We instructed annotators to use pre-coordinated concepts wherever possible (Figure 1).
3. **No meta-annotations.** Clinical annotations are sometimes enriched by qualifiers called meta-annotations, for example temporal qualifiers (is this happening now or in the past?) or negations (is this finding present or absent?). To keep the task simpler, we did not attempt meta-annotation.

Standard practice during annotation projects is to train the annotation team on the policy before evaluating their performance on a test dataset. The training and evaluation steps are repeated until consistent annotations, in line with the policy, are being produced.

Due to the complexity of the notes and terminology, the team soon discovered it was not possible to fully align annotators’ behaviors at the outset since each note contained new concepts and raised “edge cases.” The team therefore proceeded as follows:

1. Each week a new batch of notes was released to each annotator.
2. One note within each batch was shared by all annotators, enabling the Inter-Annotator Agreement (IAA) to be tracked.
3. Each week, an all-team discussion was convened to discuss progress and learnings.

Towards the end of the exercise, a selection of notes containing concepts that had been the subject of disagreement between annotators were annotated a second time. In cases of disagreement, a third round ensued, with a “best of 3” vote used to determine the correct targets.

To evaluate the IAA, we computed the Jaccard Similarity Score between the annotations produced by 2 annotators, ignoring variations in the annotation span boundaries. (In other words, the metric focused on whether annotators found the same set of clinical entities and whether they assigned them to the same concepts.) We estimate that the mean Jaccard Similarity Score across all pairs of annotators is 0.85.

Table 1 summarizes the final dataset and its splits. To our knowledge, this is the largest, publicly available dataset for real-world clinical entity linking. It has been published on PhysioNet.^11^

**Table 1.** Summary of the final annotated dataset.

Split	Number of discharge notes	Number of annotations	Number of unique concepts
Training split	204	51 572	5336
Hidden test split	68	23 234	4082
Both splits	272	74 808	6624

### Terminology

SNOMED CT (Systematized Nomenclature of Medicine Clinical Terms) is a systematically organized clinical terminology used by healthcare providers globally to facilitate the accurate recording, sharing, and analysis of clinical data. Unlike simpler medical coding systems, SNOMED CT takes a context-rich approach to organizing medical information. Features of the terminology that can be exploited by machine learning models include:

- Sets of common synonyms for each clinical term;
- A multi-hierarchy, localizing concepts in terms of their meaning;
- The ability to express any concept in terms of a unique set of “defining attributes” which, taken together, entail a precise definition.

The Entity Linking competition used the May 2023 International Edition of SNOMED CT which contains 364 901 active concepts. To simplify the exercise, only the sub-hierarchies Findings (117k concepts), Procedures (59k concepts), and Body Structures (41k concepts) were used.

### Scoring

The competition evaluated submissions’ abilities to detect the precise spans of annotated entities and link them to the correct target.

The *intersection over union* (“IoU”) measure, evaluated at the character and concept level, achieves this.

Let the indicator function $P_{d,i,c}$ take the value of 1 if the character from document d at position i is predicted to be linked to concept c, and 0 otherwise. Let the “ground truth” assignment of the same character be denoted by $G_{d,i,c}$. Then, the IoU score for concept c is given as follows:


$$\mathrm{Io}U_{c}=\frac{\sum_{d}\sum_{i}P_{d,i,c}G_{d,i,c}}{\sum_{d}\sum_{i}P_{d,i,c}\;+\;\sum_{d}\sum_{i}G_{d,i,c}}.$$

The mean IoU score used to evaluate the submission over the N documents in the test set is given as follows:


$$\mathrm{IoU}\;=\;\frac{\sum_{c}\mathrm{Io}U_{c}}{N}.$$

If penalties from misaligned span boundaries are ignored, the IoU score for a concept reduces to the Jaccard Similarity Score. Therefore, the value of the latter (0.85) for our ground truth dataset represents an *upper bound* on the best IoU score achievable by a submission.

#### Scoring example

Consider the sentence “*CT head revealed no internal hemorrhage.*” in which the concept 303653007 | Computed tomography of head (procedure) appears. The ground truth annotation links the span “*CT head*” to this concept. Suppose this were the only instance of this concept.

1. If a submission only linked the span “*CT*” to the concept, then $IoU_{303653007}=\frac{2}{7}=0.286$. The submission is penalized for the missing characters.
2. If a submission linked the span “*CT head revealed*” to the concept, then $IoU_{303653007}=\frac{2}{16}=0.125$. The submission is penalized for the extra characters.
3. If a submission linked the span “CT head” to a *different* concept, then $IoU_{303653007}=0$. (There would be a corresponding penalty for the other concept, due to the 7 extra characters that were linked to it.)

### Submissions

Further details of the winning solutions can be found in the Supplementary material.

#### First place: KIRI

The KIRI team submitted a dictionary-based approach comprising a dictionary construction component—which analyzes and expands the training data using external resources—and an annotation component—which uses the dictionary to identify spans and link them to SNOMED CT. Both components use a simple algorithm to partition clinical notes into sections and identify the headers.

#### Second place: SNOBERT

**Figure 2.**
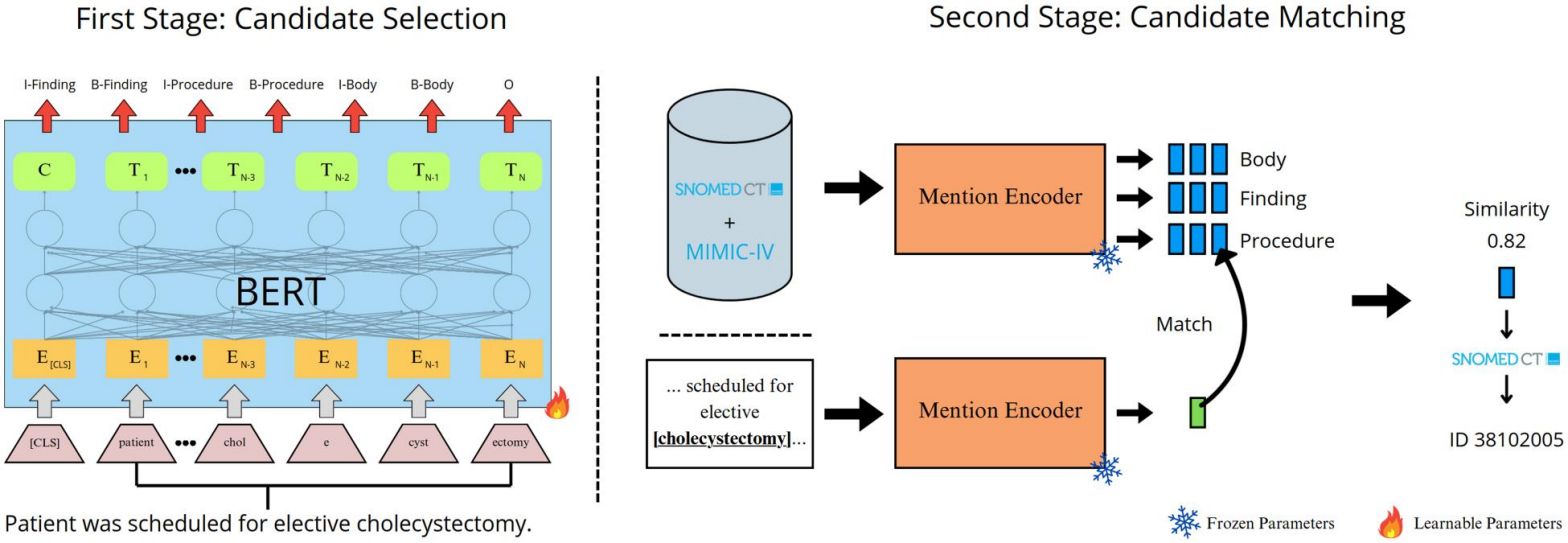
SNOBERT overview.

The SNOBERT team’s approach was based on BERT encoders. Two, finetuned BERT models were used: one for candidate selection and another for concept linking as shown in Figure 2.

#### Third place: MITEL-UNIUD

The approach devised by the MITEL-UNIUD used fine-tuned, generative pre-trained transformer models for the candidate selection and linking steps. Additionally, the linking step makes use of a retrieval-augmented-generation technique where plausible target concepts are added to a prompt, tasking the transformer to select the best match given the surrounding context.

### Calculated features for concepts

In the Results and Discussion sections, we consider 5 features of the annotations (calculated at the concept-level) which contribute to the difficulty of the entity linking task.

- **Span length**. The mean length (in characters) of all spans associated with the concept. Our hypothesis was that shorter spans would be harder to annotate. Although the evidence supports this, the effect size is small.
- **Concept entropy**. A measure of the diversity of spans associated with a concept. Modeling the bag of annotated spans associated with a concept as an approximation of a multinomial distribution, we calculate the entropy of this distribution (Figure 1C). Our hypothesis was that higher-entropy concepts would be harder to learn. The evidence supports this, although the effect size is modest.
- **Number of training examples**. We hypothesized that the more frequently a concept appeared in the training set, the better the models would be at detecting it in the test set. The evidence supports the hypothesis. Furthermore, it appears that the foundation-model–based approaches benefit more from these additional examples than does the dictionary-based approach.
- **Concept depth**. The “depth” of a concept is defined as the length of the shortest chain of “is a” relationships from the concept to the SNOMED CT “root concept.” We hypothesized that deeper concepts are more clinically nuanced and will be harder to annotate. The evidence supports this but the effect is weak.
- **Annotation entropy**. Computed as follows, for each concept C:
- Locate the set of spans, A, linked to C across the training and test sets.
- Compute the bag of concepts, B, to which the spans in A are linked.
- Modeling B as an approximation of a multinomial distribution, compute its entropy.
- The “Annotation Entropy” of a concept, C, measures the diversity of candidate concepts to which an algorithm could rationally choose to link a text span for which the ground truth is C (Figure 1D). Our hypothesis was that higher annotation entropies will be harder for the models, given the greater number of plausible targets for the linking step. Our analysis supports this.

## Results

### Comparative analysis of the winning solutions

The official results for the winners and the mean IoU scores were as follows:

1. KIRI—0.4202
2. SNOBERT—0.4194
3. MITEL-UNIUD—0.3777

The median score among all the final submissions was 0.2572, with scores ranging from 0.006 to 0.4202.

While competition scoring rules determined a definitive winner, bootstrap resampling of the IoU scores (n = 1000) the first 3 entries in Table 2 showed that the 95% confidence intervals for KIRI and SNOBERT overlap, indicating the difference between their performances was not statistically significant at *α *= 0.05.

**Table 2.** Bootstrapped resampling of IoU scores and class-weighted IoU scores for the winning teams.

*Bootstrapped resampling of IoU scores for the winning teams*			
**Team**	**Bootstrap mean**	**Lower confidence level (2.5%)**	**Upper confidence level (97.5%)**
KIRI	0.420	0.408	0.433
SNOBERT	0.419	0.407	0.432
MITEL-UNIUD	0.378	0.366	0.391
***Bootstrapped resampling of class-weighted IoU scores for the winning teams***			
KIRI	0.619	0.570	0.669
SNOBERT	0.615	0.570	0.661
MITEL-UNIUD	0.572	0.530	0.620

A shortcoming of the mean, per-concept IoU measure is that no accounting is made for the number of instances of a concept: failing to annotate 100 out of 100 instances results in the same penalty as failing to annotate 1 out of 1 instance.

An alternative metric, the class-weighted IoU, accounts for the number of occurrences of each concept. Table 2 also shows a bootstrapped estimate of class-weighted IoU scores. All scores have increased, indicating that rare concepts are harder than common concepts for all 3 approaches.

Some 1288 concepts appeared in the test set only. There were 1946 associated annotations (8.4% of all test-time annotations). Perhaps unsurprisingly, concepts not appearing in the training set were significantly harder to annotate than those which did (the winners’ IoUs for the former were 0.270, 0.235, and 0.231; for the latter, they were 0.526, 0.521, and 0.470). We note that the dictionary-based approach is better at detecting test-time only concepts (IoU 0.270) than are the deep learning approaches.

Finally, we measure the association between the concept-level IoU scores and the calculated features of each concept using Kendall’s Tau. For the Span Length, the Tau values were 0.05, 0.04, and 0.03; for the Concept entropy, 0.23, 0.26, and 0.24; for the number of training examples, 0.25, 0.29, and 0.28; and for concept depth, −0.06, −0.05, and −0.06.

**Figure 3.**
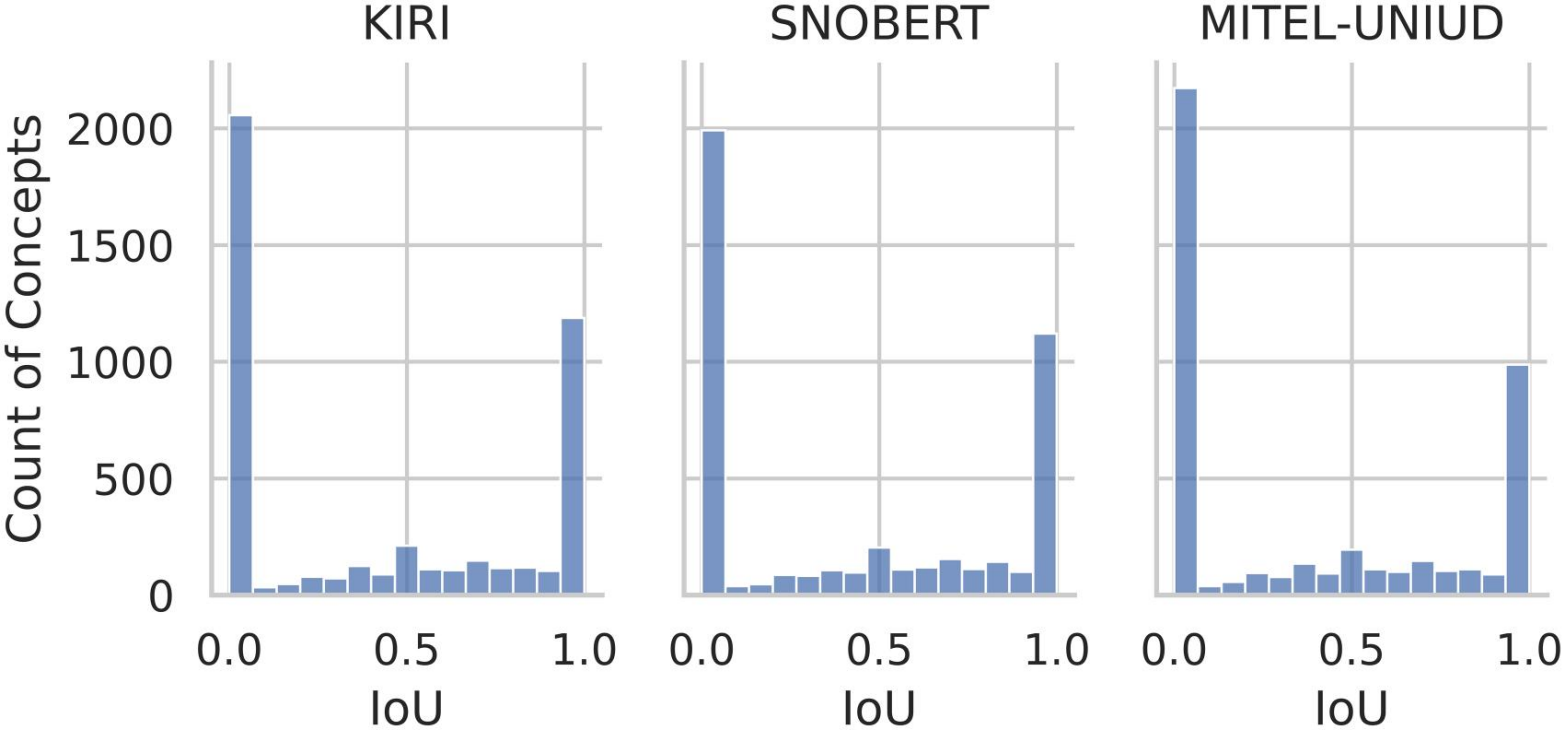
Histogram of per-concept IoU scores for each team.

Figure 3 shows histograms of the per-concept IoU distributions for the winners. All are strongly bimodal, with many concepts having scores < 0.1, and many with scores > 0.9.

Examining the pairwise correlation of concept-level IoU scores between each pair of winners using the Spearman’s rank method, we obtain values of 0.75, 0.71, and 0.74, indicating that a given concept will tend to be of similar difficulty for each model.

### Voting classifier

Finally, we implement a “majority voting classifier” using the winning submissions. The IoU of this classifier is 0.442—a modest improvement on the winning submission. The weighted IoU score is 0.626—showing very little improvement on the weighted IoU of the winning submission. This result is not unexpected, given the strong association between per-concept IoUs identified above.

## Discussion

### Analysis of low-scoring concepts

To determine the underlying reasons for the notable correlation in per-concept IoU scores, we divided the 6624 unique concepts into a “hard” set (the 1260 concepts with an IoU < 0.1 for all submissions) and an “easy” set (the 5364 concepts with an IoU ≥ 0.1 for at least one submission).

We fitted a gradient boosting algorithm to the calculated set of concept features, with the objective of learning to classify whether a concept would be easy or hard. We randomly sampled 4968 (75%) concepts for training and evaluated the classifier on the remaining 1656 (25%) concepts. The classifier achieved a class-weighted F1 Score of 0.81 on the held-out set.

**Figure 4.**
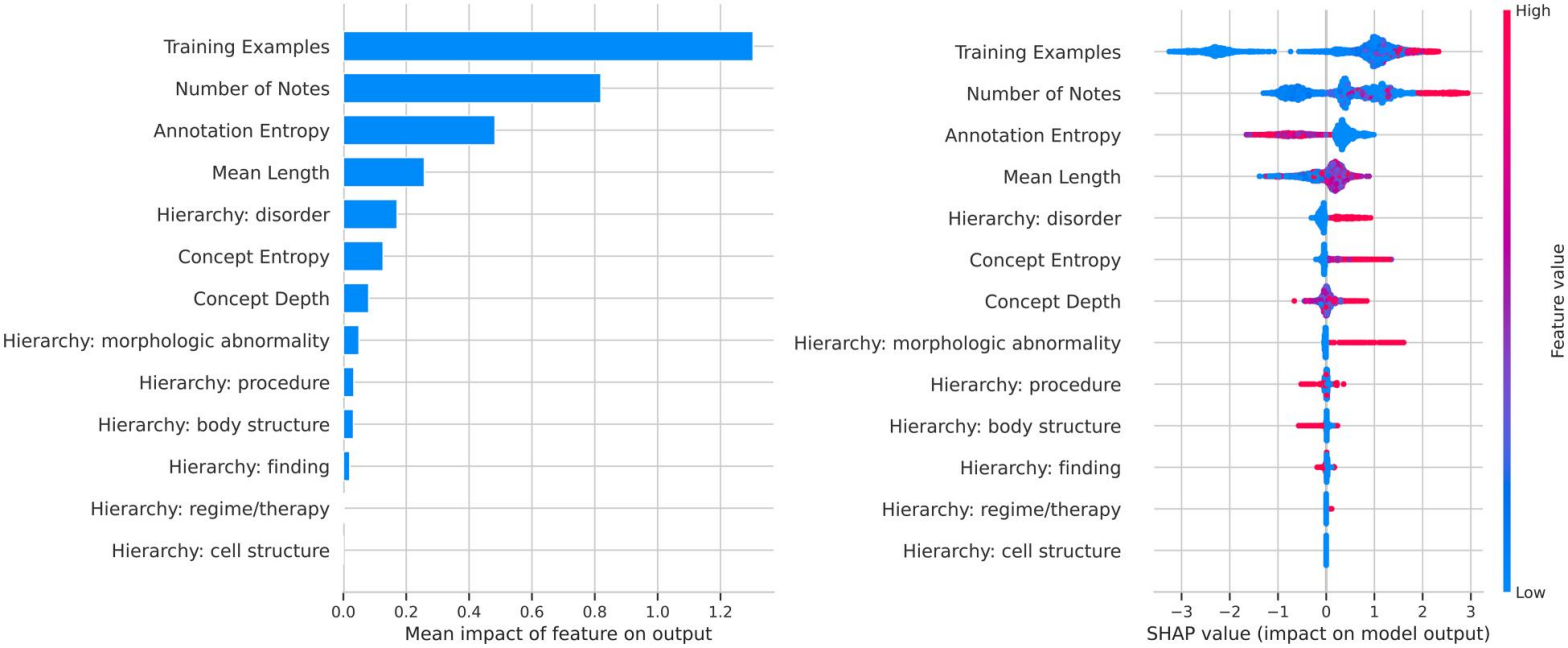
SHAP value analysis.

We performed an analysis of SHAP values^12^ for the classifier. Figure 4 shows the results of an analysis of feature importance using SHAP values and the per-example contribution (positive or negative) of high and low SHAP values to the difficulty of the entity linking task.

**Figure 5.**
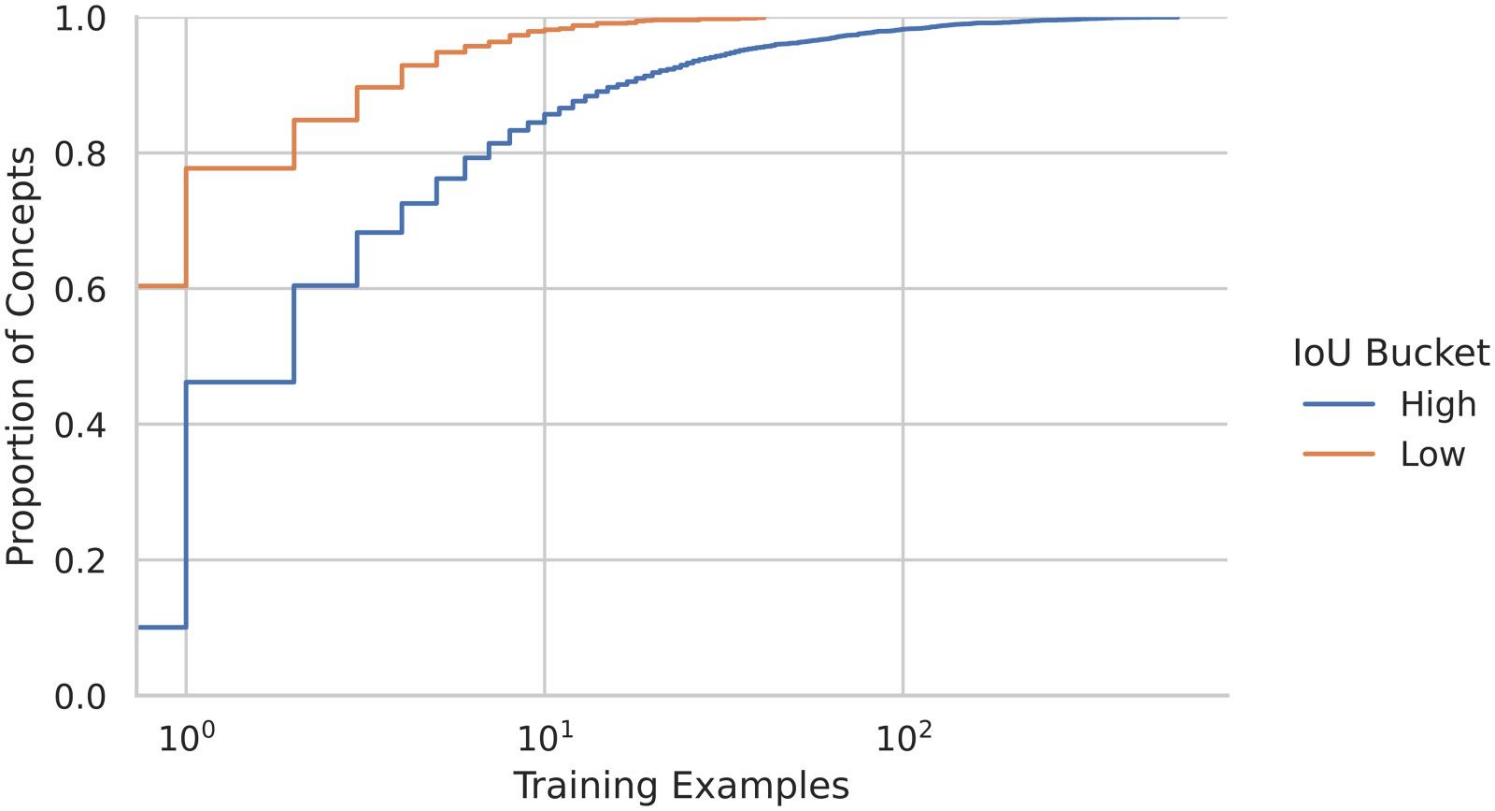
Empirical CDF showing number of training examples for low-IoU and high-IoU concepts.

The number of training examples has by far the greatest effect on the difficulty; concepts with few training examples are strongly associated with low-IoU concepts. The point is underscored in Figure 5.

The second most impactful feature is the number of notes in which the concept appears. Although this feature is correlated with the number of examples, dropping it from the classifier reduces the F1 score to 0.78, indicating that increased note diversity reduces annotation difficulty. We hypothesize that concepts appearing in more clinical notes offer Entity Linking models a greater variety of surrounding contexts, enabling them to better distinguish and link the concept.

The third most important feature is the Annotation Entropy. Concepts associated with spans which are themselves associated with many other concepts are much more likely to have low IoU scores than those which do not.

We hypothesize that 2 factors drive this effect:

1. High annotation entropies indicate that a linker has a lot of “choice” when choosing targets. Rather than rely on the span, linkers must consider the surrounding context. During training, the linker will often see only a few examples, making it difficult to learn which features of the context are salient.
2. There are entities in the data where different annotators have selected different target concepts. If so, the context holds no clue as to the “correct” concept and models will simply predict the modal target, a policy which will yield an IoU score of 0 on the (incorrect) concept and a significant “false-positive” penalty for the modal target concept.

This is illustrated with an example of a concept that all models failed to annotate: 162498009 | Symptom not changed (finding)

This concept appeared 20 times across the dataset. The following spans were linked to the concept: “*stable*” (x15), “*similar to previous examination*” (x1), “*no substantial change*” (x1), “*no notable interval change*” (x2), and “*no resolution of symptoms*” (x1).

This set of spans, in turn, was linked to SNOMED concepts 238 times in the data. A breakdown is given in Table 3, showing that:

**Table 3.** Illustrated example: concepts linked to any of the spans: “stable,” “similar to previous examination,” “no substantial change,” “no notable interval change,” or “no resolution of symptoms.”

Concept	Occurrences	Mean IoU (winning submissions)	Mean precision	Mean recall
72970002 | Normal vital	5	0.91	0.97	0.94
signs (finding)				
162498009 | Symptom not	20	0.00	0.00	0.00
changed (finding)				
271651005 | Stable blood	6	0.39	1.00	0.39
pressure (finding)				
359746009 | Patient’s	206	0.60	0.63	0.94
condition stable (finding)				
359748005 | Patient	1	0.09	0.38	0.14
condition unchanged				
(finding)

1. All target concepts are semantically similar. Several could be used interchangeably without affecting interpretation. Semantically similar concepts are hard for both models and humans to disambiguate.
2. The concept 359746009 | Patient’s condition stable (finding) is the modal target for these spans; therefore, entity linkers will have a strong prior for linking to it. The evidence supports this: all submissions show near perfect recall for this concept but lower precision.
3. The target 72970002 | Normal vital signs (finding) only appears in the “labs results” sections of the notes, which is highly structured. The high precision and recall scores indicate that structured context is easily learned.

### Failure modes for low-IoU concepts

The per-concept character-level IoU score used in the competition is related to the character-level Precision and Recall scores:


$$\mathrm{Io}U_{c}=\frac{\mathrm{Precisio}n_{c}\;\times \mathrm{Recal}l_{c}}{\mathrm{Precisio}n_{c}+\mathrm{Recal}l_{c}-\mathrm{Precisio}n_{c}\;\times \mathrm{Recal}l_{c}}$$

The precision score for a concept, *c*, is affected by 2 kinds of error:

- **False-Positive Link**: the linker step selects *c* as a target for a span which should have been linked to another concept.
- **False-Positive Span**: the entity recognition step flags a span which is not associated with an annotation in the ground truth dataset, and the linker links it to *c*.

Likewise, recall scores are also affected by 2 kinds of error:

- **False-Negative Link**: the linker step selects another concept as a target for a span when it should have selected *c*.
- **False-Negative Span**: the entity recognition step misses an annotation linked to *c* entirely.

(Note that a single error by the linker generates both a False-Positive Link error and a False-Negative Link error, affecting 2 concepts.)

We studied which kinds of errors the algorithms are making. Starting with the set of ground truth annotations from the test set, *G*, and the bag union of all predicted annotations from the winning submissions, *S*, we use Algorithm 1 to calculate the error types.

Algorithm 1: Algorithm to calculate the error types Results ←∅

**FOR**  g ∈G:

  **FOR**  s ∈S:

   **IF**  Overlaps(g, s):

    **If**  Concept(g)=Concept(s):

     Results←Results+(g, Correct)

   **ELSE**:

     Results←Results+(g, False Negative Link)

     Results←Results+(s, False Positive Link)

   **ELSE**:

    Results←Results+ (g, False Negative Span)

**FOR**  s ∈S:

  **FOR**  g ∈G:

   **IF**not  Overlaps(s,g):

    Results←Results+(s, False Positive Span)

Table 4 contains a count of the number of characters affected by each of the error types above on the 1260 low-IoU concepts.

**Table 4.** Error categories (across all submissions).

Annotation category	Correct	False-Positive Link	False-Positive Span	False-Negative Link	False-Negative Span
“Hard”	0.2%	9.9%	5.2%	41.9%	43.0%
“Easy”	63.8%	9.6%	9.7%	7.1%	9.8%

Table 4 makes it clear that most of the errors for “hard” concepts are recall-related, roughly balanced between False-Negative Link errors and False-Negative Span errors.

Concepts with high False-Negative Span errors are being missed by the entity recognition step. A detailed analysis revealed that over 90% of these errors are associated with just 263 concepts; only 4 of which have more than 5 training examples.

We conclude that all of the entity linking models struggle to detect clinical entities where they have seen fewer than 5 such examples in the training data. This problem is particularly acute where the span is a relatively common string.

**Figure 6.**
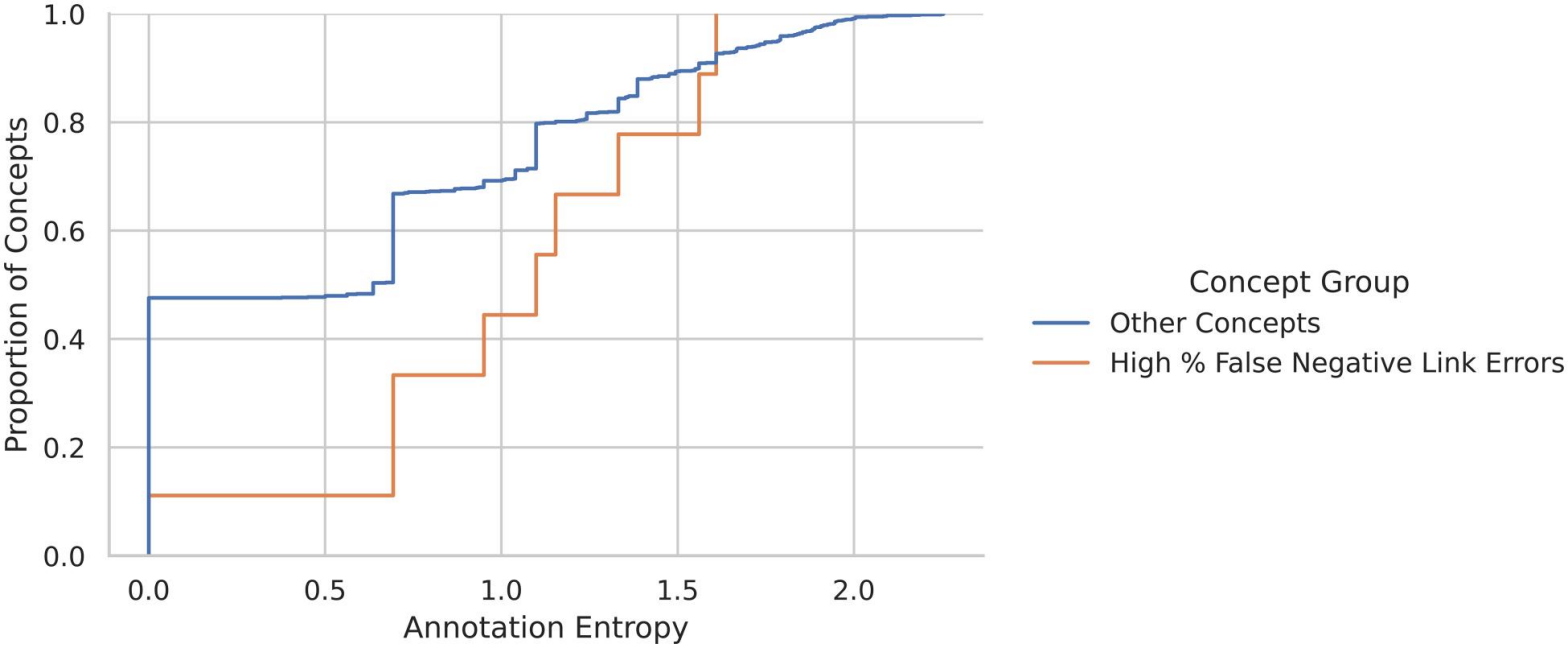
Empirical CDFs of annotation string entropies associated with concepts experiencing high Recall Type A errors compared to other concepts.

Concepts with a high percentage of False-Negative Link errors are being mis-classified by the linker, which is selecting other (incorrect) targets instead. There are 288 concepts associated with 90% of these errors. Figure 6 shows the distribution of annotation entropies associated with these concepts, as compared to other concepts. They are significantly higher, indicating that the cues in the surrounding context are being missed, likely a consequence of the fact that it is difficult to learn such cues when few examples of a (span, concept) pair exist in the training data. Instead, the models are likely linking to the most common target for a given span.

## Conclusion

We ran an open competition to evaluate machine-led approaches for entity linking on a large and diverse dataset of real-world clinical data.

We learned that human-led annotation projects are difficult when the task involves linking source data containing reams of clinical short-hand and localized abbreviations to a large and nuanced target terminology. Widely accepted clinical annotation guidelines rely on a training phase in which annotator behavior is aligned, before the full annotation task begins. We discovered that, in a complex annotation scenario like ours, new “edge cases” and new concepts are encountered throughout the project, necessitating an ongoing process of re-aligning the annotation policy and reviewing past work.

To raise the bar for large-scale annotation efforts like ours, we advocate for the development of tools and techniques for monitoring annotation quality as well as for detecting and remediating potential inconsistencies. Some recent work has begun to explore this.^13^

Our contribution includes what is, to our knowledge, the largest publicly available clinical annotation dataset. Our analysis suggests that expanding this dataset to increase the number of examples of “low-frequency” concepts will benefit entity linking models. Adding meta-annotations and post-coordination (omitted due to time and budget constraints) would bring these closer to immediate clinical utility. Ironically, given the quantity of patient notes in the world, this is a field starved of data.

The winning submissions represent 3 very different approaches to entity linking. Our comparative analysis highlighted few meaningful distinctions in the strengths and weaknesses of each approach, indicating that data—rather than model architecture—is the limiting factor on performance.

None of the submissions could, at present, be considered usable for clinical decision support. However, with 25% of concepts having IoU scores in excess of 0.95 across all winning submissions, the problem appears tractable with modern methods.

Our analysis suggests that the technical key to high-performing entity linkers lies in extracting the most distinguishing and relevant elements of the surrounding context when the distribution of examples is heavy-tailed. Future work should focus on methods for learning salient context features from sparse examples.

### Lessons for annotating with SNOMED CT

1. **“Absent” findings are less useful**. A small proportion of clinical findings have SNOMED concepts denoting both their presence and their absence, for example 60728008 | Swollen abdomen (finding) vs 300405003 | Normal abdominal contour (finding). When annotating clinical documents, this leads to confusion, with annotators able to choose between the “present” concept plus a meta-annotation to signal absence or simply using the “absent” concept. We believe the former policy is better since it can be uniformly applied across all Finding annotations. A similar argument applies to bilateral body structures, where “left” and “right” would better be kept as meta-annotations, rather than being subsumed into concepts in some cases but not others.
2. **Post-coordination**. Post-coordination is a powerful tool for constructing clinical concepts which do not exist in a terminology. In practice, the main use of the Body Structure hierarchy is to facilitate post-coordination. Therefore, annotation guidelines should stipulate that Body Structure entities are annotated only when a corresponding Finding or Procedure annotation exists, to add clinical meaning to the annotation.
3. **Discontiguous spans**. Consider the sentence “*Family History of cancer—lung and liver.*” Our annotation guidelines precluded overlapping spans; therefore, the concept 93870000 | Malignant neoplasm of liver (disorder) would have to link to the span “*liver*” only. It is then not clear whether the concept 363358000 | Malignant neoplasm of lung (disorder) should be linked to “*cancer—lung*” or simply to “*lung*.” Situations like this are a source of inconsistency in our annotations, leading to “high annotation entropy.” A better solution would be to allow the entire span “*cancer—lung and liver*” to be linked to both concepts.

## Supplementary Material

ocaf104_Supplementary_Data

## Data Availability

The annotated dataset used in this study is publicly available on PhysioNet at https://doi.org/10.13026/s48e-sp45. It is derived from the MIMIC-IV-Note corpus (version 2.2), which is accessible to credentialed researchers through PhysioNet (https://physionet.org/content/mimiciv-note/2.2/) subject to completion of appropriate data use agreements and training.
