# Supplementary material for: SNOMED CT entity linking challenge
Source: J Am Med Inform Assoc. 2025 Jul 14;32(9):1397–406. doi: 10.1093/jamia/ocaf104 (PMC12361850; doi:10.1093/jamia/ocaf104)
Supplement: ocaf104_Supplementary_Data [file ocaf104_supplementary_data.docx]

# Appendix

### The KIRI solution

The KIRI team submitted a dictionary-based approach comprising a *dictionary construction* component - which analyses and expands the training data using external resources - and an *annotation component* that uses the dictionary to identify spans and link them to SNOMED CT. Both components use a simple algorithm to partition clinical notes into sections and identify the headers.

### Dictionary construction component

The dictionary construction component constructs a mapping $(S,M)\to C$ where $S$ is a section header, $M$ is a “mention” (annotated span) and $C$ is a SNOMED CT concept. Each dictionary entry implies that if span $M$ is found within section $S$ it should be mapped to concept $C$.

There are two main sources of data for the initial construction of the dictionary: the training annotations and SNOMED CT itself - which associates text “synonyms” to each code. (Other, open-source vocabularies, do the same - often in specific domains).

Training examples are added to the dictionary as follows: for every annotation in the training set that matches mention $M$ with code $C$, the section $S$ in which $M$ resides is identified. Then, a mapping $(S,M)\to C$ is added to the dictionary - unless $(S,M)$ is in the “blacklist”. The blacklist contains substrings which appear frequently across multiple sections. Each blacklisted entry is duplicated, replacing $S$ with the wildcard value (“*”), implying it can be matched in a variety of sections.

Then, each dictionary entry is evaluated against the training data: whenever $M$ is found in a section $S$ we record whether or not $M$ was indeed annotated and associated with $C$. The rate of correct annotation is recorded as the entry’s precision score. In following pruning step, low precision entries are removed.

The sections to which wildcard entries of the form $(*,M)\to C$ can be matched are determined as follows:

1. In SNOMED CT, each concept is associated with a “hierarchy” $H$ (e.g. “procedure”). For a given section $S$, we collect all concepts appearing in this section across all notes.
2. We extract $H(S)$, the set of all the hierarchy categories associated with these codes.

This set limits the wildcard entries that can be matched in $S$. Therefore, an entry $(*,M)\to C$ can only be matched to text $M$ in section $S$ if $H$ is in $H(S)$.

Next, we generate dictionary entries from SNOMED CT and additional vocabularies, such as ICD10 and READ. SNOMED concepts are associated with textual synonyms describing their meanings. We map the terms from the additional vocabularies to SNOMED CT vis OHDSI standard mappings [16].

Initially, each vocabulary term was added to the wildcard section “any”, with the following pre-processing rules:

1. Texts were normalised to deal with brackets and parentheses
2. Only terms of 5 words or less were allowed
3. Only terms of more than 3 characters were allowed

Finally, variations of the above terms were added as well - including all permutations of word orders, rephrasing of terms and some word replacements. Although these variations are often nonsensical, their inclusion does not hamper annotation quality since they are unlikely to appear in the clinical notes.

### Annotation component

The annotation component processes documents as follows:

1. Partition the document into sections;
2. For each section $S$, get the dictionary entries for that section
3. For each such entry, $(S,M)\to C$, if $M$ matches a span in $S$, annotate it with $C$
4. Resolve multiple annotations of the same span
5. Refine annotations based on SNOMED CT relationships

Matching mentions to dictionary entries is done while ignoring case, whitespace and special characters. (An exception: case is not ignored if the mention $M$ is a single, all-caps word.) The letter “*s*” is allowed to appear at the end of the span, to allow for matches in the plural form.

Multiple annotations of the same span are resolved by prioritising annotations for longer spans over shorter spans and preferring mentions from section-specific entries over those paired with the “any” section header.

In a post-processing step, we attempt to extend each span by searching for preceding ‘attribute’ words, such as “*left*”, “*upper*”, “*mild*” or “*acute*”. SNOMED CT relation- ships are used to extract pairs of concepts with an ‘is-a’ relation, while their descriptions differ by a single word. Then, if the shorter-description concept was annotated and the attribute word is found in the preceding text, the span is extended and mapped to the longer-description concept.

### A.2 The SNOBERT solution

The approach taken by the SNOBERT team (documented in [17]) consists of two stages: Candidate Selection and Candidate Matching. Figure 3 illustrates the process.

**FIGURE 3**: SNOBERT overview

***Alt text:*** *An illustration of the two stages of the SNOBERT algorithm. The first stage, “Candidate Selection”, demonstrates how a BERT model is used to encode the tokens in an input sentence into named entity types, for example “Finding” and “Procedure”. The second stage illustrates how the SAPBERT encoder is used. The SNOMED CT concepts were vectorised and used as a reference set. Then, each of the “mentions” extracted in the first stage were encoded and matched to the nearest concept using the Cosine distance.*

In the first stage, the NER classification problem was solved, and in the second stage, for each classified span from the first stage, the corresponding concept ID in SNOMED terminology was linked.

SNOBERT utilized the NER pipeline LabelStudio [18] to address certain annotation inaccuracies, such as those caused by shifts due to tags. Approximately 10 notes out of 204 underwent corrections, involving adjustments to around 150 annotation IDs. These corrections specifically targeted errors resulting from shifted annotations. Furthermore, most annotated notes were missing some labels in the paragraphs with the following headers: ’medications on admission:,’ ’\_\_\_ on admission:,’ ’discharge medications:.’ We excluded these parts from the training process. All HTML markup elements, such as the line break element (’br’) or the new line (’n’), were removed from the notes.

A static dictionary of the most common concepts from training data was generated and matched with test data in the post-processing step using a string-matching search. The team applied "one-to-one" matching, linking only complete coincidences.

### First Stage: Candidate Selection

The concepts were initially separated according to the first-level hierarchy (the first entry in the path to the concept) according to SNOMED terminology. Thus, the classes "Finding," "Procedure," "Body structure," and "None" could be emphasized. During Tokenization, the words got flags in "B-I-O" tagging format: "B" (Beginning) means the first token of the first word within an annotation; "I" (Inside) - is the first token of a subsequent word within an annotation; "O" (Outside) - is as a stand-alone token or single word token. Consequently, we have seven classes: "I-Finding," "B-Finding," "I-Procedure," "B-Procedure," "I-Body," "B-Body," and "O".

### Second Stage: Candidate Matching

To link classified terms from the first stage, we match first-stage embeddings with embeddings of terms from SNOMED CT by cosine similarity. For this purpose, the whole database extracted concepts from the "Body structure," "Findings," and "Procedure" paths, which are about 200k unique concepts with the Mention Encoder. In the solution, the SapBERT [19] model was applied.

### Training

We employed 4-fold Cross-Validation with each fold consisting of 51 discharge notes. For the first stage, we used a domain-specific model for Biomedical Natural Language Processing [20], pretrained on abstracts from PubMed and full-text articles from PubMed-Central. The base version, microsoft/BiomedNLP-BiomedBERT-base-uncased-abstract-fulltext, was used. Using early stopping criteria, 75 epochs on average were needed for each split. We used the ADAM optimizer with a learning rate of 3×10^(-5), batch size of 8, and class weighting. Training took 30 minutes on 4 GPUs (NVIDIA A100-SXM4-40GB). We found a slight improvement of 0.0005 in IoU when applying the Masked Language Model (MLM) pre-training technique. To accomplish this, we used microsoft/BiomedNLP- BiomedBERT-large-uncased-abstract weights as an initial model. This optional pre- training step took 24 hours on 4 GPUs.

### A.3 The MITEL-UNIUD solution

The approach devised by the MITEL-UNIUD used fine-tuned, generative pre-trained transformer models for both the candidate selection and the linking steps. Additionally, the linking step makes use of retrieval-augmented-generation techniques, selecting plausible candidates for inclusion into the transformer’s context prior to prompting it to select a target concept.

### Entity Recognition

The entity recognition process is a critical initial phase in the MITEL-UNIUD team’s approach to the SNOMED CT Entity Linking Challenge. This phase is designed to ensure high accuracy in identifying medical terms from clinical notes, which are crucial for effective medical documentation processing. It is composed of the following steps:

1. **Text Segmentation**. The process begins with the segmentation of clinical text into chunks. This segmentation is strategic, allowing for the application of the model to text that is narrow and contextually homogeneous, which is essential for accurate term extraction. The clinical notes are divided in such a way that each chunk contains enough context to be standalone while being small enough to manage effectively.
2. **Model Application**. The team employs the Mistral-7B-Instruct-v0.2 [14] model, which was fine-tuned on a subset of the training data to optimise its performance for this specific task. This model is developed at handling the complexity of medical terminology and the context in which these terms are used. To accommodate the variability in the length of terms and their context within the notes, two versions of the model are used: one is optimised for shorter chunks of 100 tokens, suitable for extracting shorter, more straightforward medical terms; the other is adjusted for longer chunks of 500 tokens, ideal for more complex terms or terms used in extended contexts.
3. **Dual-Model Strategy**. After the initial text segmentation, each chunk is processed by the appropriate model based on its length. This dual-model strategy allows the team to leverage the strengths of both models, optimising the extraction process across a range of medical documentation styles and lengths. It effectively addresses the challenges posed by different types of medical language usage, from concise to verbose.
4. **Annotation and Combination**. Following the application of the models, the terms identified within each text chunk are annotated by the model. These annotations mark the specific spans of text that correspond to medical terms. Once annotation is complete, a phase follows in order to combine the results from the two models. This combination is not merely aggregative; it involves a mechanism to resolve potential overlaps. When the same or similar terms are identified in overlapping segments by both models, the longer, more comprehensive annotation is retained. This ensures that the most detailed and contextually appropriate annotations are used.

This entity recognition subsystem sets a foundation for the challenge’s requirements, effectively handling the complexities of clinical text and setting the stage for accurate medical terminology coding.

### SNOMED-CT Coding

Following the precise identification of medical terms through the entity recognition phase, the SNOMED CT coding process in the MITEL-UNIUD solution is designed to classify these terms according to the SNOMED CT standards. This stage is crucial for converting raw annotated text into standardised medical codes.

1. **Integration of FAISS for Document Retrieval**. The first step in the coding process leverages the FAISS (Facebook AI Similarity Search) vector database [15]. This tool is employed to facilitate the retrieval of documents and terms relevant to the annotated medical terms extracted during entity recognition. The FAISS index is specially augmented with a detailed mapping of terms to their respective SNOMED CT codes. This mapping includes not only the terms but also their synonyms and related medical terminology, which enhances the accuracy and relevance of the retrieval process.
2. **Dual-Phase Classification Approach**. Once the relevant documents and terms are retrieved via FAISS, the process moves to the initial classification phase. In this phase, each annotated term from the entity recognition phase is matched against the retrieved terms to find the closest SNOMED CT code. This step is critical as it narrows down the possible codes from the vast array of SNOMED CT options, focusing only on the most likely candidates. After the initial matching, the second phase of classification begins. This phase takes into account the broader context in which each term appears, including nearby text and the specific section of the document. The refined context helps to clarify ambiguities and ensure that the chosen SNOMED CT code accurately reflects the clinical intent of the term. For example, the same term may have different codes depending on whether it is used in a diagnostic, therapeutic, or descriptive manner.
3. **Multi-Faceted Model Integration**. To further enhance the classification accuracy, the system integrates multiple contextual elements within the LLM. This integration involves considering the specific term to be classified, its surrounding text for con- text, the section title of the document, and insights from the top document chunks retrieved from the FAISS index. This multi-layered approach ensures a compre- hensive understanding of each term’s usage, significantly improving the precision of the final code assignment.
4. **Optimization Techniques**. The coding phase also employs a series of optimization techniques to refine the output further. Terms that are either irrelevant or outside the scope of the training dataset are excluded using a "remove list," while important but overlooked terms are added using an "add list." These lists help to fine-tune the final annotations by removing noise and ensuring completeness. As the system processes more documents, it continuously learns and adjusts the FAISS index and the term mapping strategies to improve both retrieval and classification accuracy over time.

This approach to coding ensures that every identified term is accurately coded, reflecting its proper medical significance, and supports the broader goals of enhancing healthcare documentation and analytics.
